# Use of feature importance statistics to accurately predict asthma attacks using machine learning: A cross-sectional cohort study of the US population

**DOI:** 10.1371/journal.pone.0288903

**Published:** 2023-11-22

**Authors:** Alexander A. Huang, Samuel Y. Huang

**Affiliations:** 1 Northwestern University Feinberg School of Medicine, Chicago, IL, United States of America; 2 Virginia Commonwealth University School of Medicine, Richmond, VA, United States of America; Tehran University of Medical Sciences, ISLAMIC REPUBLIC OF IRAN

## Abstract

**Background:**

Asthma attacks are a major cause of morbidity and mortality in vulnerable populations, and identification of associations with asthma attacks is necessary to improve public awareness and the timely delivery of medical interventions.

**Objective:**

The study aimed to identify feature importance of factors associated with asthma in a representative population of US adults.

**Methods:**

A cross-sectional analysis was conducted using a modern, nationally representative cohort, the National Health and Nutrition Examination Surveys (NHANES 2017–2020). All adult patients greater than 18 years of age (total of 7,922 individuals) with information on asthma attacks were included in the study. Univariable regression was used to identify significant nutritional covariates to be included in a machine learning model and feature importance was reported. The acquisition and analysis of the data were authorized by the National Center for Health Statistics Ethics Review Board.

**Results:**

7,922 patients met the inclusion criteria in this study. The machine learning model had 55 out of a total of 680 features that were found to be significant on univariate analysis (P<0.0001 used). In the XGBoost model the model had an Area Under the Receiver Operator Characteristic Curve (AUROC) = 0.737, Sensitivity = 0.960, NPV = 0.967. The top five highest ranked features by gain, a measure of the percentage contribution of the covariate to the overall model prediction, were Octanoic Acid intake as a Saturated Fatty Acid (SFA) (gm) (Gain = 8.8%), Eosinophil percent (Gain = 7.9%), BMXHIP–Hip Circumference (cm) (Gain = 7.2%), BMXHT–standing height (cm) (Gain = 6.2%) and HS C-Reactive Protein (mg/L) (Gain 6.1%).

**Conclusion:**

Machine Learning models can additionally offer feature importance and additional statistics to help identify associations with asthma attacks.

## Introduction

Asthma is a prevalent chronic respiratory disease that heterogeneously affects approximately 300 million people worldwide, causing recurrent episodes of coughing, wheezing, shortness of breath, and chest tightness [1]. Asthma attacks can be triggered by various environmental and genetic factors, including allergens, air pollution, stress, and infections [2]. These attacks can be debilitating, leading to hospitalization and even death in severe cases [3]. Moreover, asthma can significantly affect an individual’s quality of life, limiting their physical activities, causing sleep disturbance, and impairing their emotional well-being [4].

Asthma self-management plans aim to prevent attacks by helping patients identify when their asthma control is deteriorating and modifying their treatment accordingly [5]. However, predicting the risk of an asthma attack is currently a largely qualitative process, and there is no widely used algorithm to help identify patients at risk [6]. Most studies on the subject have used univariate regression modeling to identify risk factors for asthma attacks, which do not determine the predictive performance of an optimal combination of factors in individual patients [7–15].

To address these limitations, machine learning can be employed to identify factors that can cause asthma attacks. In this study, we used the National Health and Nutrition Examination Surveys (NHANES), a robust sample, to assess asthma attacks using an enhanced questionnaire. By employing XGBoost, which has strong backing from medical literature supporting it as a predictive machine learning model and a transparent machine learning process by the name of Shapely Additive Explanations (SHAP), our findings will identify hidden patterns and relationships that may not be evident through traditional statistical methods and provide valuable insights into the risk factors for asthma attacks and inform the development of personalized treatment plans for asthma patients [16–18]. Shapley Additive Explanations (SHAP) is a feature importance method that provides insights into the contribution of individual features in predicting a target variable. It offers a unified framework for understanding the importance of features in a machine learning model, regardless of their correlation or dependence. When it comes to handling highly correlated variables, SHAP considers the collective contribution of features rather than attributing importance solely to individual variables. SHAP assigns each feature a Shapley value, which represents the average marginal contribution of a feature across all possible feature combinations. This approach helps address the issue of redundant or highly correlated variables. In the case of highly correlated variables, SHAP considers their joint contribution rather than duplicating importance [19]. The Shapley values are designed to allocate credit appropriately among the correlated features based on their actual impact on the model’s predictions. If two variables are highly correlated but provide similar information, their individual Shapley values may be reduced to reflect their shared contribution, rather than ranking both variables as highly important independently [20]. Through this approach, the study aimed to contribute to the broader understanding of asthma and facilitate improved management strategies for this condition.

## Methods

The complex, multi-stage, cross-sectional cohort study utilized data from the publicly available National Health and Nutrition Examination Survey (NHANES) and included participants who completed questionnaire covering demographic information, dietary habits, exercise routines, and mental health, laboratory tests and physical exams. Participants were recruited for the NHANES program through a complicated, multistage probability sampling design. A stratified, multistage area probability design was used to select a representative sample from the civilian, non-institutionalized U.S. population for the sampling plan. Oversampling certain subgroups (such as low-income individuals, racial and ethnic minorities, and older adults) to obtain more precise estimates for these groups was done as part of the NHANES sampling design to ensure that the sample was representative of the U.S. population. Household interviews and in-person physical examinations in mobile examination centers were used for recruitment. The National Center for Health Statistics (NCHS) Ethics Review Board provided permission for the study’s data collection and analysis. Before analysis, all data, including medical records, survey responses, and demographic information, were de-identified to ensure participant anonymity. Authors did not have access to information that could identify participants during or after data collection. Furthermore, all participants provided verbal consent for their data to be made public.

### Dataset and cohort selection

The NHANES 2017–2020 was developed by the NCHS, and it has been used to assess the health and nutritional status of the American population. To gather information regarding health, nutrition, and physical activity for the NHANES dataset, the Centers for Disease Control and Prevention (CDC) conducted a series of intricate, cross-sectional, multi-stage surveys on a nationally representative cohort of the population of the United States. Patients in the NHANES dataset who were adults (those over the age of 18) and who had completed the demographic, dietary, exercise, and mental health questionnaires as well as data from their physical and laboratory examinations were the subjects of our investigation.

### Assessment of asthma attacks

The enhanced questionnaire used in NHANES asks patients about their experience with asthma over the past 12 months. Specifically, patients were asked whether they had experienced an episode of asthma or an asthma attack during this time period. Patients were asked “During the past 12 months, (have you/has SP) had an episode of asthma or an asthma attack?

### Independent variable

In NHANES, the demographics, dietary, physical examination, laboratory, and medical questionnaire datasets contained potential model covariates. The NHANES dataset yielded a total of 680 covariates. An indicator of having experienced an asthma attack within the previous year was combined with all covariates.

### Model construction and statistical analysis

Univariate logistic models used self-reported asthma attacks as the outcome to find covariates associated with asthma attacks. On univariate analysis, the final machine learning model had covariates with a p-value less than 0.0001. An initial filter on the dataset made use of univariable logistic models to make sure that each of the 680 covariates used in the machine learning models were strong independent covariates. Due to this initial filtering, physicians were also able to determine whether risk factors were clinically relevant. After performing initial filtering, relevant risk factors were identified by utilizing model importance statistics derived from machine-learning models.

The machine learning model XGBoost was used because of its widespread use in the literature and its improved predictive accuracy for healthcare predictions. Other studies using the NHANES cohort found that XGBoost was the most effective, offering the best balance between training efficiency, model accuracy, and transparency. A train:test set was used to calculate the final set of model fit parameters (80:20). In this study, the model fit parameters used were the area under the receiver operator characteristic curve (AUROC), sensitivity, specificity, positive predictive value, negative predictive value, prevalence, detection rate, detection prevalence, and balanced accuracy.

### Model feature importance statistics and SHAP visualization

Model covariates were ranked according to Gain, Cover, and Frequency to identify factors that were associated with having an asthma attack within the previous year. The Gain is the feature’s relative contribution to the model. The total number of observations made regarding this feature is referred to as the Cover. The Frequency is the percentage of times a feature appears in the trees of the machine-learning model. The Gain statistic was chosen as the method for ranking features according to importance because it is easy to understand: the proportion of the final prediction that the covariate influenced. The continuous covariates with the strongest relationship between the potential risk factors and having an asthmatic attack within the past year were visualized using SHAP explanations.

## Results

Table 1 shows the 7,922 patients that met the inclusion criteria in this study. Individuals reported mean Octanoic Acid intake as a Saturated Fatty Acid (SFA) of 0.42 grams a day in the general population (SD = 0.49), 0.54 grams a day among those that had an asthmatic attack (SD = 1.05), and 0.41 grams a day among those that did not have an asthmatic attack (SD = 0.44). Individuals had eosinophil percents of 2.78% (SD = 2.08) among the general population, 3.36% (SD = 2.73) among those that had an asthmatic attack, and 2.76% (SD = 2.05) among those that did not have an asthmatic attack. Individuals had an average hip circumference of 107.57 cm (SD = 14.89) among the general population, 115.16 cm (SD = 19.99) among those that had an asthmatic attack, and 107.24 cm (SD = 14.54) among those that did not have an asthmatic attack. Individuals had a standing height on average of 167.11 cm (SD = 9.95) among the general population, 164.73 cm (SD = 9.60) among those that had an asthmatic attack, and 167.22 cm (SD = 9.95) among those that did not have an asthmatic attack. Individuals had on average a HS C-Reactive protein level of 4.09 mg/L (SD = 7.85) among the general population, 6.08 mg/L (SD = 7.90) among those that had an asthmatic attack, and 4.00 mg/L (SD = 7.83) among those that did not have an asthmatic attack.

**Table 1:** Demographic variables.

	Total	Had Asthma Attack	Did Not have Asthma Attack	p-value
Total	7922	338	7584	
Eosinophils percent (%)	2.78 (2.08)	3.36 (2.73)	2.76 (2.05)	p<0.001
Eosinophils number (1000 cells/uL)	0.20 (0.17)	0.25 (0.27)	0.19 (0.16)	p<0.001
Red cell distribution width (%)	13.90 (1.39)	14.27 (1.78)	13.88 (1.36)	p<0.001
HS C-Reactive Protein (mg/L)	4.09 (7.85)	6.08 (7.90)	4.00 (7.83)	p<0.001
Albumin, refrigerated serum (g/dL)	4.06 (0.35)	3.94 (0.36)	4.06 (0.35)	p<0.001
Albumin, refrigerated serum (g/L)	40.59 (3.50)	39.43 (3.64)	40.64 (3.49)	p<0.001
BMXWT—Weight (kg)	84.02 (23.31)	92.21 (29.58)	83.65 (22.93)	p<0.001
BMXHT—Standing Height (cm)	167.11 (9.95)	164.73 (9.60)	167.22 (9.95)	p<0.001
BMXBMI—Body Mass Index (kg/m**2)	30.00 (7.65)	33.99 (10.64)	29.82 (7.44)	p<0.001
BMXARMC—Arm Circumference (cm)	33.72 (5.42)	35.56 (6.53)	33.64 (5.35)	p<0.001
BMXWAIST—Waist Circumference (cm)	100.65 (17.47)	107.84 (21.18)	100.34 (17.23)	p<0.001
BMXHIP—Hip Circumference (cm)	107.57 (14.89)	115.16 (19.99)	107.24 (14.54)	p<0.001
General Health Condition	2.77 (1.03)	3.30 (1.04)	2.74 (1.02)	p<0.001
Routine Healthcare Location	1.18 (0.42)	1.07 (0.25)	1.18 (0.43)	p<0.001
Hospitalization	1.89 (0.34)	1.80 (0.40)	1.89 (0.33)	p<0.001
Patient Reported Presence of Mental Health Issue	1.89 (0.31)	1.76 (0.43)	1.90 (0.30)	p<0.001
Household Food Security	1.70 (1.03)	2.00 (1.18)	1.69 (1.02)	p<0.001
Adult Food Security	1.69 (1.03)	2.01 (1.20)	1.68 (1.02)	p<0.001
Told Doctor they had sleeping disorder	1.71 (0.47)	1.49 (0.65)	1.72 (0.46)	p<0.001
How Often the Patient Felt Overly Sleepy during the Day	1.80 (1.19)	2.17 (1.25)	1.78 (1.18)	p<0.001
Height_inches	66.43 (4.17)	65.19 (3.98)	66.48 (4.17)	p<0.001
Self View of Weight	1.90 (0.99)	1.63 (1.04)	1.91 (0.99)	p<0.001
WHD080Q Ate Fruits and Vegetables	0.26 (0.44)	0.37 (0.48)	0.26 (0.44)	p<0.001
WHD080S Ate less sugar, candy, sweets	0.23 (0.42)	0.33 (0.47)	0.22 (0.42)	p<0.001
WHD080T Ate less junk food	0.24 (0.43)	0.35 (0.48)	0.23 (0.42)	p<0.001
PHQ_2	0.72 (1.31)	1.26 (1.60)	0.70 (1.29)	p<0.001
PHQ_9	3.18 (4.30)	5.38 (5.18)	3.08 (4.23)	p<0.001
Having Little Interest in Doing Things	0.37 (0.77)	0.60 (0.89)	0.36 (0.76)	p<0.001
Feeling Down, Depressed, or Hopeless	0.35 (0.73)	0.66 (0.93)	0.34 (0.72)	p<0.001
Trouble Sleeping or Sleeping too Much	0.64 (0.98)	1.11 (1.21)	0.62 (0.96)	p<0.001
Feeling Tired or Having Little Energy	0.74 (0.95)	1.12 (1.03)	0.72 (0.94)	p<0.001
Poor Appetite or Overeating	0.40 (0.81)	0.69 (0.97)	0.39 (0.80)	p<0.001
Trouble Concentrating on Things	0.26 (0.69)	0.46 (0.86)	0.26 (0.68)	p<0.001
Moving or Speaking Slowly or too Fast	0.16 (0.56)	0.33 (0.73)	0.15 (0.55)	p<0.001
DPQ100—Difficulty These problems have caused	0.21 (0.52)	0.41 (0.70)	0.20 (0.51)	p<0.001
phq_2_cutoff	0.05 (0.22)	0.10 (0.31)	0.05 (0.21)	p<0.001
PHQ_9_cutoff	0.07 (0.25)	0.16 (0.36)	0.07 (0.25)	p<0.001
Gender	1.51 (0.50)	1.70 (0.46)	1.50 (0.50)	p<0.001
SFA 8.0 Octanoic (gm)	0.42 (0.49)	0.54 (1.05)	0.41 (0.44)	p<0.001
Median CAP, decibels per meter (dB/m)	264.02 (63.31)	280.23 (67.55)	263.31 (63.02)	p<0.001
Gender.1	1.51 (0.50)	1.70 (0.46)	1.50 (0.50)	p<0.001
routine_healthcare	0.83 (0.37)	0.93 (0.25)	0.83 (0.38)	p<0.001
hospitalization	0.11 (0.32)	0.20 (0.40)	0.11 (0.31)	p<0.001
mental_health	0.11 (0.31)	0.24 (0.43)	0.10 (0.30)	p<0.001

Descriptive statistics for demographic characteristics and all covariates within the machine learning model, stratified by whether patients reported an asthma attack or not.

**Fig 1:**
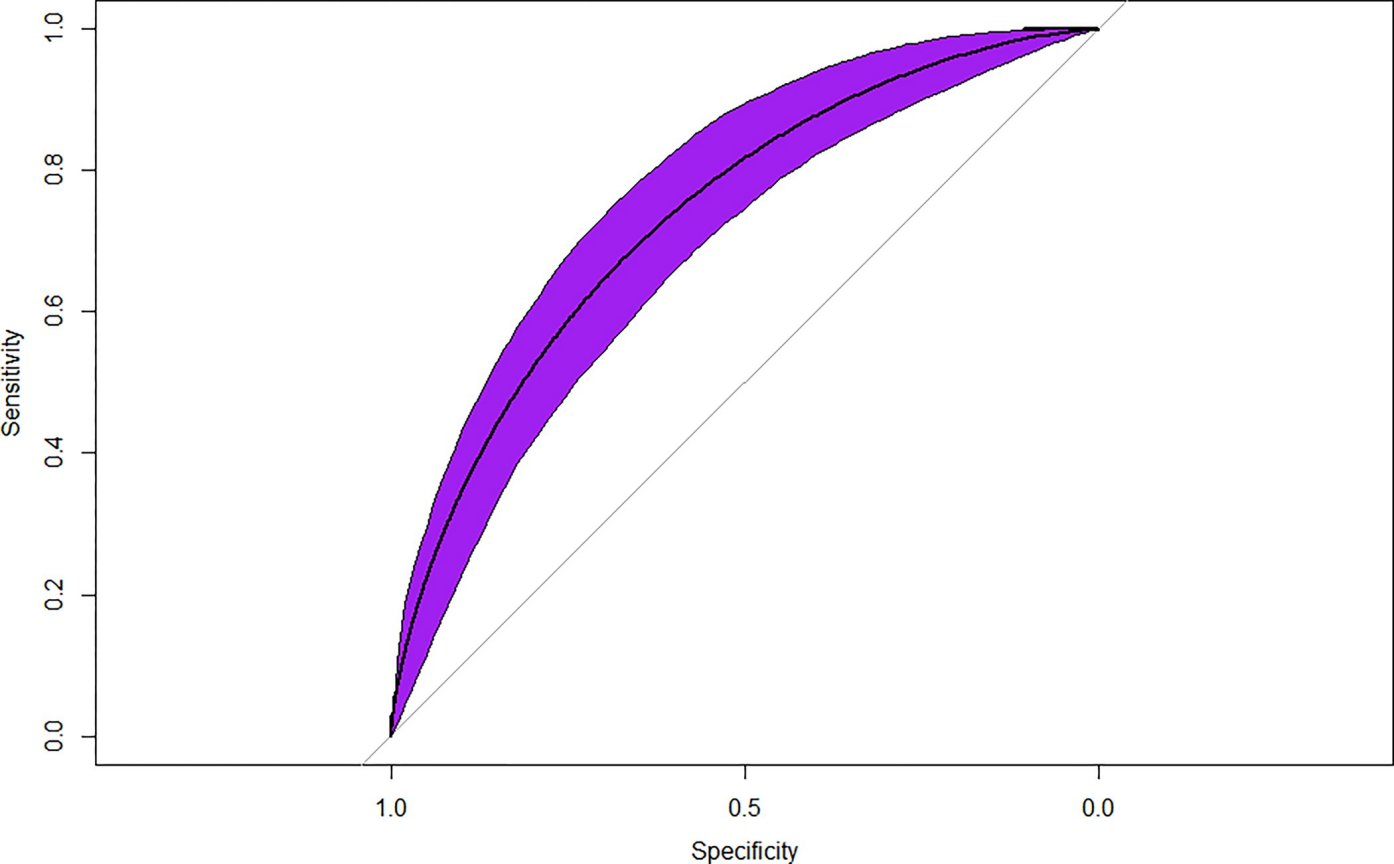
Receiver operator characteristic curve and model statistics. The Receiver operating characteristic curve for the machine-learning model predicting whether the patient had an asthma attack within the past year or not. AUROC = 0.737 (P<0.001).

The machine learning model had 55 out of a total 680 features that were found to be significant on univariate analysis (P<0.0001 used). These were fitted into the XGBoost model and Fig 1 shows an AUROC = 0.737. Table 2 shows Sensitivity = 0.960, Specificity = 0.225 were observed. Table 3 shows that the top five highest ranked features by gain, a measure of the percentage contribution of the covariate to the overall model prediction, were Octanoic Acid intake as a SFA (gm) (Gain = 8.8%), Eosinophil percent (Gain = 7.9%), BMXHIP–Hip Circumference (cm) (Gain = 7.2%), BMXHT–standing height (cm) (Gain = 6.2%) and HS C-Reactive Protein (mg/L) (Gain 6.1%).

**Table 2:** Model metrics.

Sensitivity	0.96
Specificity	0.225
Positive predictive value	0.0302
Negative predictive value	0.967
Prevalence	0.025
Detection rate	0.024
Detection prevalence	0.025
Balanced accuracy	0.592

Table 2 displays the performance metrics of a statistical model. The model’s predictive accuracy is evaluated using various measures such as sensitivity, specificity, positive predictive value (PPV), negative predictive value (NPV), Prevalence, Detection Rate, Detection Prevalence, and Balanced Accuracy.

**Table 3:** Model gain statistics.

Feature	Gain	Cover	Frequency
SFA 8.0 Octanoic (gm)	0.088	0.084	0.098
Eosinophils percent (%)	0.079	0.080	0.082
BMXHIP—Hip Circumference (cm)	0.072	0.108	0.068
BMXHT—Standing Height (cm)	0.062	0.051	0.066
HS C-Reactive Protein (mg/L)	0.061	0.029	0.061
BMXBMI—Body Mass Index (kg/m**2)	0.052	0.066	0.048
BMXARMC—Arm Circumference (cm)	0.047	0.038	0.048
BMXWAIST—Waist Circumference (cm)	0.045	0.019	0.049
Median CAP, decibels per meter (dB/m)	0.041	0.016	0.050
Told Doctor they had sleeping disorder	0.040	0.082	0.018
BMXWT—Weight (kg)	0.039	0.032	0.038
PHQ_9	0.036	0.075	0.030
Red cell distribution width (%)	0.034	0.046	0.045
Albumin, refrigerated serum (g/dL)	0.031	0.047	0.038
MCQ520—Abdominal pain during past 12 months?	0.031	0.045	0.018
Height_inches	0.025	0.015	0.022
Eosinophils number (1000 cells/uL)	0.021	0.023	0.020
General Health Condition	0.017	0.016	0.014
weight_loss_10lbs	0.016	0.006	0.014
Adult Food Security	0.015	0.017	0.015
Patient Reported Presence of Mental Health Issue	0.014	0.012	0.013
Gender	0.014	0.011	0.010
Trouble Sleeping or Sleeping too Much	0.012	0.010	0.012
Feeling Tired or Having Little Energy	0.009	0.002	0.009
How Often the Patient Felt Overly Sleepy during the Day	0.008	0.004	0.009
DPQ100—Difficulty These problems have caused	0.008	0.010	0.009
Routine Healthcare Location	0.007	0.007	0.005
MCQ053—Taking treatment for anemia/past 3 mos	0.007	0.007	0.007
Household Food Security	0.007	0.002	0.009
Ate Fruits and Vegetables	0.006	0.003	0.005
Ate_less_sugar	0.006	0.003	0.005
MCQ160a - Doctor ever said you had arthritis	0.005	0.001	0.005
Feeling Down, Depressed, or Hopeless	0.005	0.004	0.006
general_health	0.004	0.001	0.005
MCQ160b - Ever told had congestive heart failure	0.004	0.003	0.005
Poor Appetite or Overeating	0.004	0.001	0.005
Moving or Speaking Slowly or too Fast	0.004	0.003	0.005
MCQ550—Has DR ever said you have gallstones	0.004	0.001	0.005
Trouble Concentrating on Things	0.003	0.002	0.004
Ate_less_junk_food	0.003	0.001	0.003
MCQ366d - Doctor told you to reduce fat/calories	0.003	0	0.003
Hospitalization	0.003	0.001	0.003
MCQ366a - Doctor told you to control/lose weight	0.002	0.001	0.002
PHQ_2	0.002	0.001	0.003
Having Little Interest in Doing Things	0.002	0	0.002
MCQ080—Doctor ever said you were overweight	0.001	0.001	0.001
Self View of Weight	0.001	0.012	0.003
MCQ366b - Doctor told you to exercise	0.001	0	0.001

The Gain, Cover, and Frequency of all covariates within the XGBoost model. The Gain represents the relative contribution of the feature to the model and is the most important metric of model importance within this study. Covariates ordered according to the Gain statistic.

**Fig 2:**
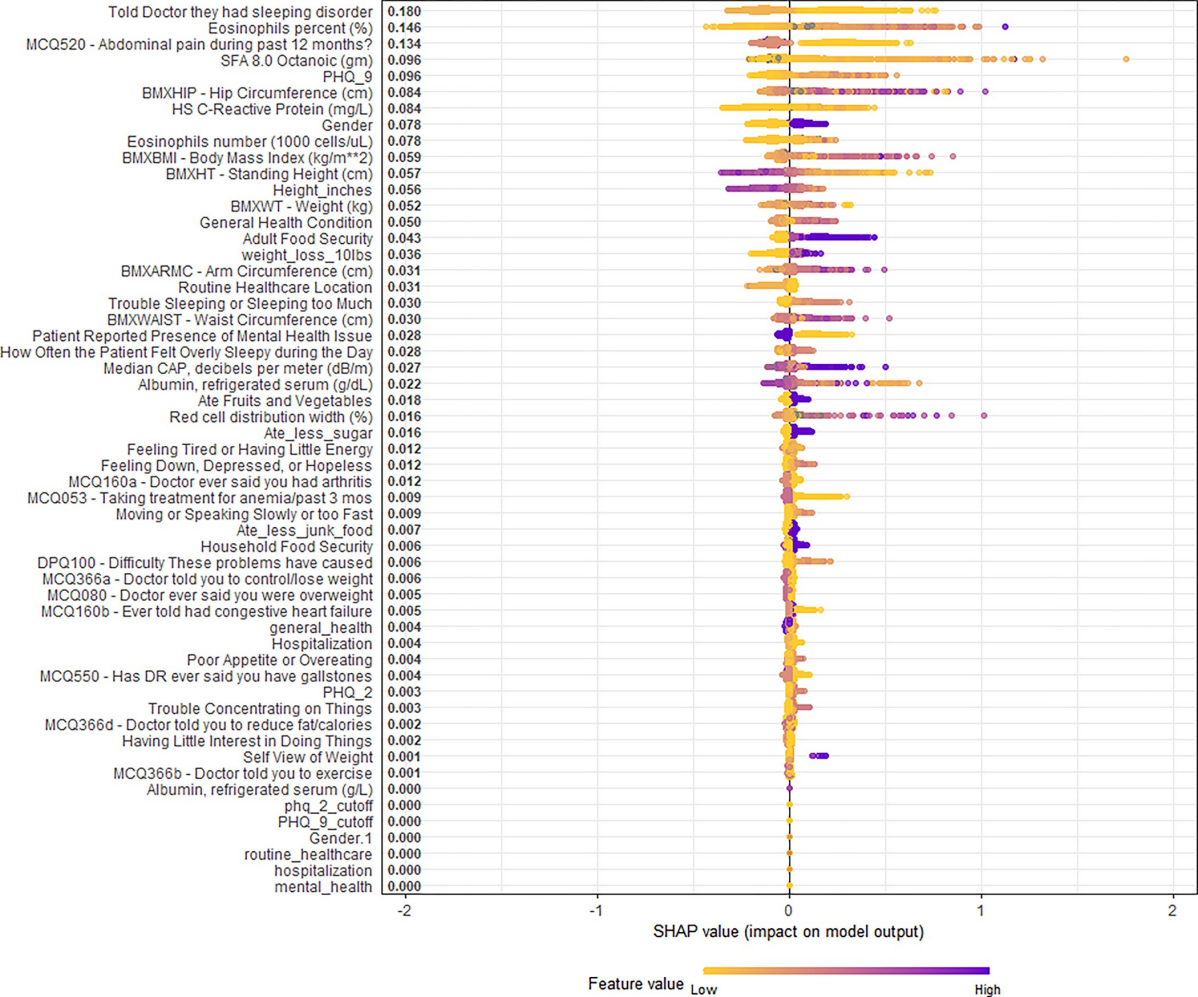
Overall SHAP explanations. SHAP explanations, purple color representing higher values of the covariate while yellow representing lower values of the covariate. X-axis is the change in log-odds for reporting an asthma attack within the past year.

In Fig 2, overall SHAP explanations can be seen for all the statistically significant covariates on univariable regression.

**Fig 3:**
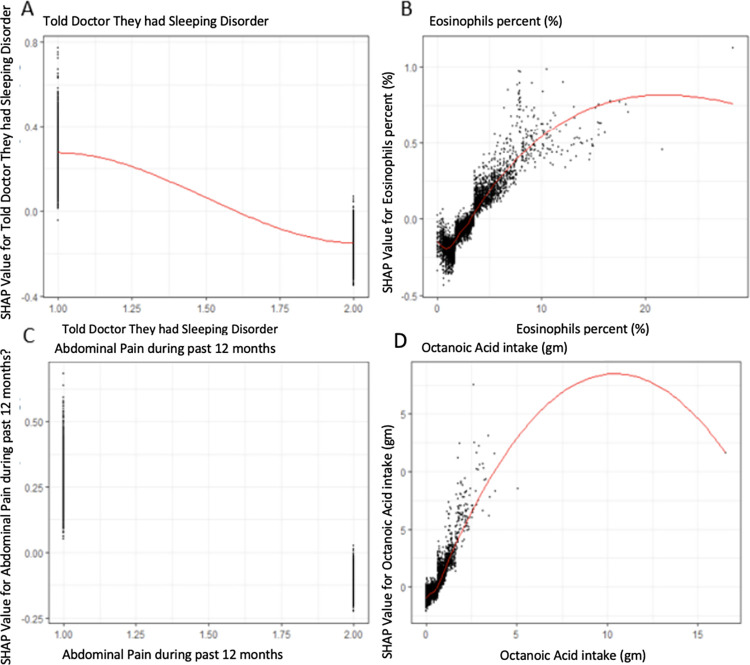
SHAP explanations for the Top 4 continuous covariates sorted by overall SHAP explanations. SHAP explanations, covariate value on the x-axis, change in log-odds on the y-axis, red line represents the relationship between the covariate and log-odds for Asthma attacks, each black dot represents an observation. Covariates: top left–Told doctor they had a sleeping disorder, top right–Eosinophil percent (%), bottom left–MCQ520 –Abdominal pain during past 12 month, bottom right–Octanoic Acid intake as a Saturated Fatty Acid (SFA) (gm).

In Fig 3 SHAP visualizations were conducted for the top four continuous covariates by overall SHAP explanations. We observed that if the patient told there doctor they had a sleeping disorder it increased their odds of having an asthmatic attack, increasing eosinophil percentage up to a asymptote of around 20% was associated with an increase risk of having an asthma attack the past year, reporting abdominal pain was associated with an increase risk of asthma attack, and increasing amounts of octanoic acid intake was associated with an increase risk of having an asthma attack within the past year.

## Discussion

In this cross sectional cohort of United States adults, a machine learning model to predict asthma attacks had great sensitivity and negative predictive value (AUROC = 0.737, sensitivity = 0.960, and NPV = 0.967) The greatest predictors for asthma attacks by SHAP explanation included if they told their doctor they had a sleeping disorder, eosinophil count, reporting abdominal pain in last 12 months, and octanoic intake in grams.

Our machine learning model contains relationships and findings that are in line with many other studies that have looked at factors associated with asthma attacks [21–25]. Our model not only combines many of the associations found in literature, but demonstrates the importance and weights each item brings to the model and accounts for linear and curvilinear relationships. In our study the top 5 characteristics sorted by gain were concordant with current literature: there is strong epidemiological evidence that diet, eosinophils, hip circumference, standing height and C-reactive protein levels are associated with asthma [22–31]. This alignment of these covariates with literature not only in statistical significance, but also in the direction of the relationships increase our confidence that the model accurately captures the physiological relationships of these factors. By using transparent machine learning tools, we can ensure that the model is detecting genuine signals within these covariates to predict asthma attacks, rather than simply replicating biases present in the dataset [32–35]. The SHAP visualizations further support the increased predictive power of these non-parametric methods by demonstrating their ability to accurately capture the non-linear interactions between covariates, without overfitting the model to achieve greater accuracy [20, 36–39].

By assisting patients in recognizing when they are increasing risk factors associated with asthma attacks, they can modify their treatment and routines with the aim to lower chances of attacks. However, there is currently no widely used guideline for identifying patients at risk, and predicting the likelihood of an asthma attack is largely a qualitative process. Many studies on the subject have used univariate regression modeling to identify asthma attack risk factors, but this method does not determine the optimal combination of factors for each patient’s predictive performance [40–42].

However, a limited number of studies have attempted to combine various risk factors to develop a risk scoring algorithm and studies are increasingly utilizing machine learning in various capacities to study asthma. One benchmark study identified patients at risk of recurrent asthma attacks using the Optimum Patient Care Research Database, identifying various predictors for attacks and evaluated them using logistic regression. The study resulted in the development of an online asthma risk prediction tool for research and clinical purposes [43–45]. However, this study only utilized logistic regression, which has been shown to be a poor classifier in cases of class imbalance.

Machine learning and artificial intelligence models have been effectively used to predict asthma attack [46, 47]. Prior studies have accurately predicted the presence of asthma attacks using machine-learning methods from a variety of datasets that consider physical parameters such as weight, blood pressure, and other metrics easily monitorable on wearable devices [48, 49]. Others look at prior exposure history as well as childhood development to predict potential of asthma exacerbation [50–53]. These studies highlight the utility of machine learning models in identifying patients at risk for asthma attacks. In the past five years, numerous machine learning studies have been conducted for asthma management, but many of these studies have been limited by small sample sizes and lack of external validation, limiting their generalizability [47]. Our study, in contrast, utilized a large cohort, providing a more robust foundation for generalization and application of the developed algorithms. What our study adds to the literature is a large dataset (N = 7,922) that contains well representative data of US adults that looks at how a variety of nutrition and health examination data contribute to prediction of asthma attacks.

Many studies have also been pushing for increased transparency in modeling in general, but especially so in the field of pulmonology. Our study utilizes a novel paradigm recently described in literature that combines the commonly used XGBoost algorithm with SHAP to describe how the model is predicting each feature and how important it is [20].

The primary advantage of using this algorithmic method for covariate identification is the ability to systematically search through many potential factors, without relying on researcher judgment that may be influenced by personal biases [47]. This approach also enables the ranking of covariate importance using gain, cover and frequency statistics, allowing us to estimate the relative contribution of each covariate to asthma attack risk [20]. Additionally, SHAP visualizations can be employed after covariate selection and model building to ensure that each factor aligns with current literature on its association with asthma attacks, or to investigate any discrepancies and evaluate potential data-quality errors [18]. This approach strengthens the reliability and accuracy of our findings. The conclusion of the study highlights the potential of machine learning models in identifying strong and hidden associations related to predicting asthma attacks in patients within a one-year timeframe. By utilizing machine learning techniques, researchers can uncover patterns and relationships in the data that may not be immediately apparent through traditional statistical approaches. These models have the ability to analyze various factors and their interplay to provide insights into the occurrence of asthma attacks.

### Limitations

Limitations of this machine-learning analysis include the cross-sectional nature and its ability to introduce bias. However, this was addressed by using training and testing sets to minimize overfitting. Additionally, SHAP visualizations provide a means for researchers to assess the plausibility of each covariate and determine whether the effects are due to true signal or noise that could contribute to a type-1 error. After considering the strengths and weaknesses of these methods, we propose that machine-learning can serve as a valuable initial step in identifying potential risk factors, which can then be further evaluated by clinicians based on the patient’s specific clinical presentation. NHANES, while being a robust and representative dataset of the US population, lacks detailed information on the severity of asthma attacks or validation measures. The dataset primarily relies on self-reported incidents of asthma attacks without specific measures of severity. This limitation restricts the studies ability to assess the severity of asthma attacks and validate the self-reported data. However, despite this limitation, the NHANES dataset provides a comprehensive examination of various factors associated with asthma attacks and offers valuable insights into the overall burden of asthma within the population. These findings, although not capturing the full spectrum of severity or validation, can still contribute important information to guide future research and inform public health interventions.

### Conclusion

Machine learning models can be used to find strong and hidden associations for predicting if patient had asthma attack within the past year.

## Supporting information

S1 Checklist STROBE statement—checklist of items that should be included in reports of observational studies. (DOCX)

## Abbreviations

NCHS: National Center for Health Statistics
NHANES: National Health and Nutrition Examination Surveys
SHAP: Shapely Additive Explanations
